# Coronary artery bypass grafting and concomitant excision of chest wall chondrosarcoma

**DOI:** 10.1186/1749-8090-4-7

**Published:** 2009-02-18

**Authors:** Pankaj Kaul, David JR Duthie, Somsekhar Ganti, Radhika Ramnath

**Affiliations:** 1 Yorkshire Heart Centre, Leeds General Infirmary, Great George Street, Leeds, LS1 3EX, UK; 2 Department of Pathology, Saint James's University Hospital, Beckett Street, Leeds, LS9 7TF, UK

## Abstract

Coexistence of coronary artery disease and cancer with both requiring surgical treatment at the same time is rare. A 52 year male undergoing elective coronary artery bypass grafting was incidentally discovered to have a large soft tissue mass of variable consistency with cartilaginous elements arising from the right costal margin and adjoining ribs by a broad attachment and protruding into right pleural cavity. Frozen section suggested it to be either a chondrosarcoma or a teratoma. A wide excision of the mass with the adjoining muscle and periosteum along with quadruple coronary artery bypass grafting was done. This report is unusual on account of a) being the first reported case in world literature of concomitant excision of chondrosarcoma and coronary artery bypass grafting and b) the conservative management of the incidentally discovered chondrosarcoma by wide excision rather than chest wall resection with no local recurrence to date. Pathology of chondrosarcoma, in particular, and various management strategies when coronary artery disease and cancer coexist, in general, is discussed.

## Case presentation

**Figure 1:**
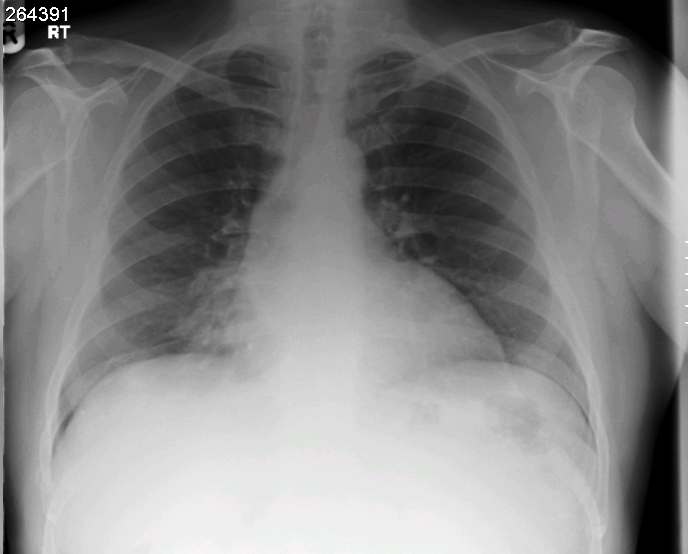
**Chest radiograph showing a soft lobular paracardiac shadow in right pericardiophrenic angle**.

**Figure 2:**
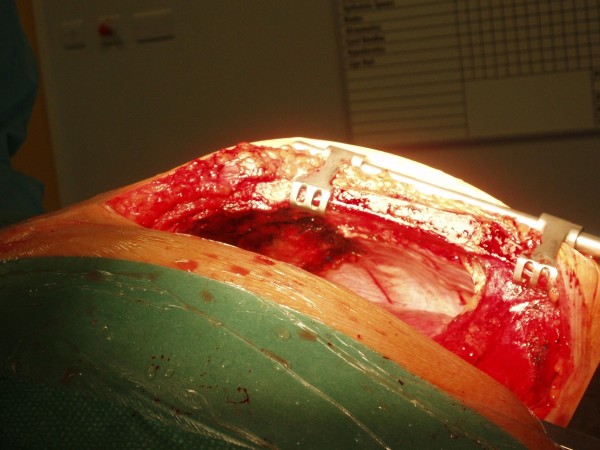
**Intraoperative photograph showing large mass arising from inner surface of right costal margin and the adjacent ribs and protruding into right pleural cavity (arrow)**.

**Figure 3:**
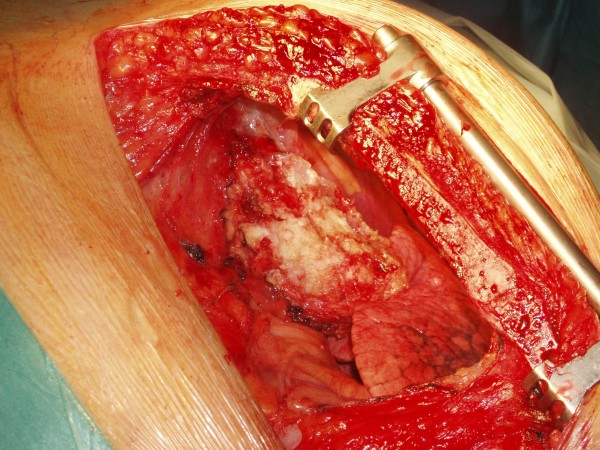
**Intraoperative photograph showing the mass having been excised from the costal margin but still attached to the pleural remnants**.

**Figure 4:**
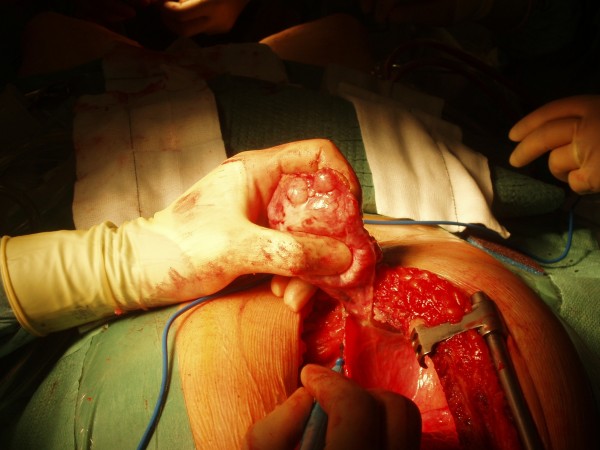
**Intraoperative photograph showing the knobbly mass of uneven consistency being excised from last pleural remnants**.

**Figure 5:**
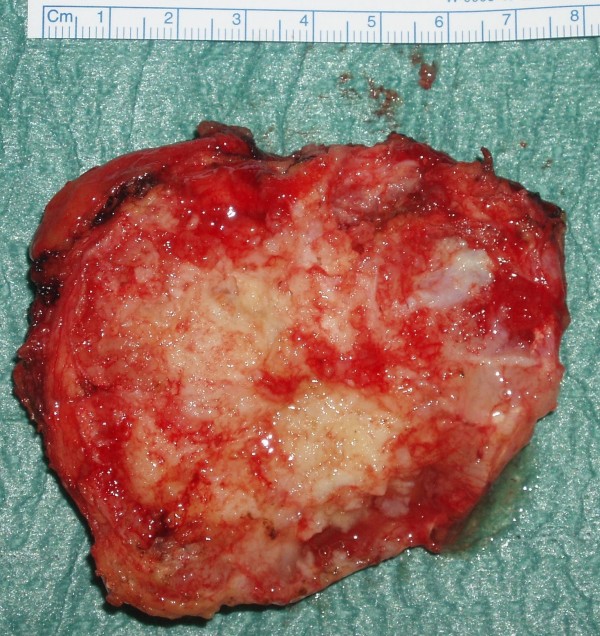
**The excised tumour mass showing its irregular variegated costochondral surface interspersed with cystic and solid areas**.

A 52 year old male smoker undergoing coronary artery bypass grafting for three vessel coronary artery disease and moderately impaired left ventricular function was felt to have a mass arising from the under surface of right costal margin adjacent to right lower sternal margin while sternopericardial ligament was being broken off by finger dissection prior to sternotomy. Preoperative chest X-ray suggested a soft, globular paracardiac shadow in relation to the right pericardiophrenic angle, appreciated better retrospectively (Figure 1) Sternotomy was made, the right pleura was opened to facilitate delineation of the mass. The mass measured 8 × 6 × 3 cms and was arising from the right costal margin and the adjacent surfaces of 7^th^, 8^th^, 9^th ^and 10^th ^ribs (Figure 2). The mass was of firm to hard and variable consistency and was filled with cartilaginous material and there was no definite demarcation between the mass and the chest wall. The chest wall mass was excised in its entirety along with the intercostal muscle and the periosteum (Figure 3, Figure 4 and Figure 5). The frozen section revealed it to be either a chondrosarcoma or teratoma. Quadruple coronary artery bypass grafting to left anterior descending artery and its diagonal branch, obtuse marginal branch of circumflex artery and left ventricular branch of right coronary artery was performed using left internal mammary artery and long saphenous vein for conduits, employing cardiopulmonary bypass with antegrade cold blood cardioplegic arrest. Patient made uncomplicated postoperative recovery.

**Figure 6:**
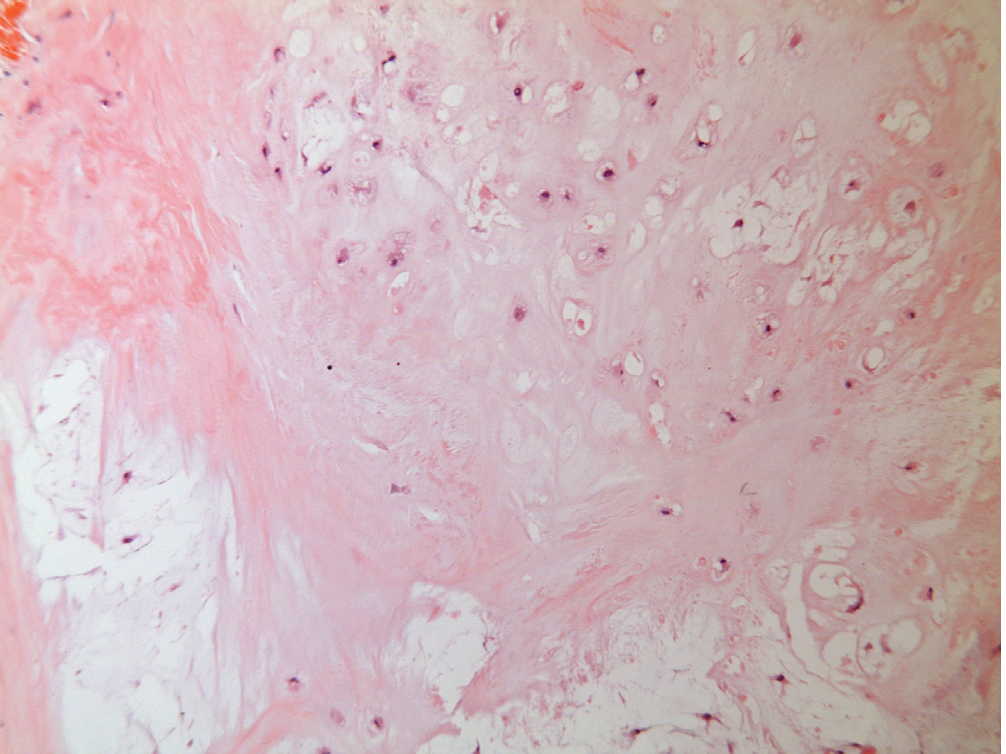
**Histopathology picture (low power) showing grade 1 chondrosarcoma with lobules of cartilage separated by fibrous tissue**.

**Figure 7:**
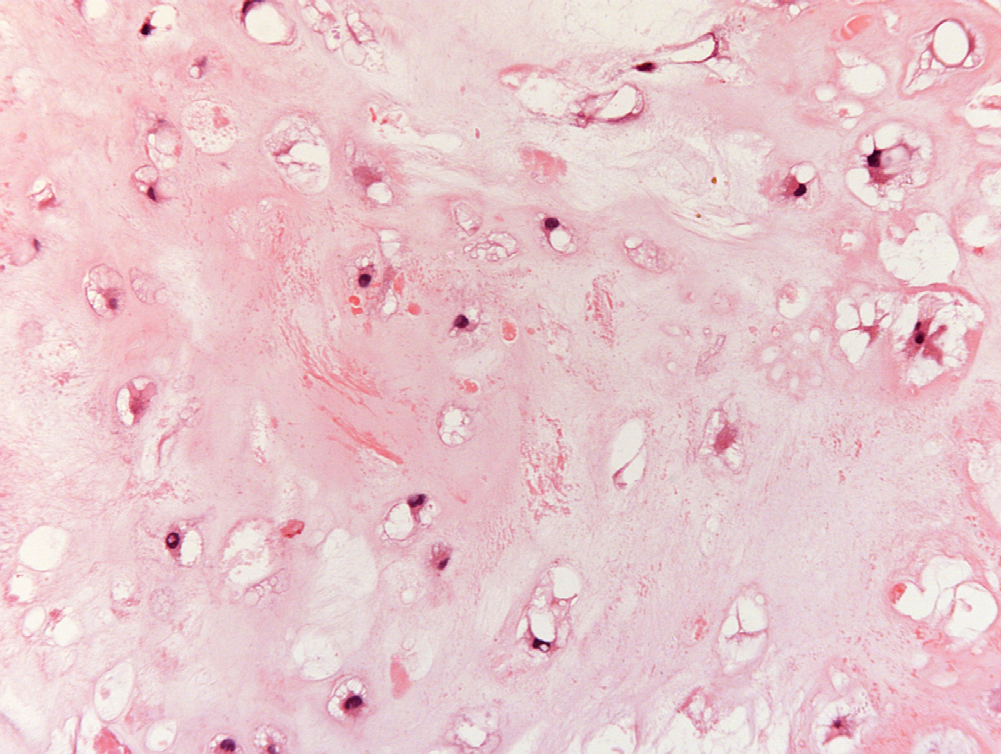
**Histopathology picture (high power) showing lobules of cartilage composed of atypical chondrocytes**.

Histopathology revealed the overall appearances of grade 1 chondrosarcoma with a tumour composed of lobules of cartilage of varying size separated by fibrous tissue (Figure 6). The chondrocytes showed cytological atypia, binucleate cells, focal hypercellular areas with areas of calcification and necrosis (Figure 7). The tumour was seen to focally infiltrate into surrounding skeletal muscle.

The patient was discussed at the Regional Sarcoma MDT. Further excisional surgery in view of the positive surgical margins was not felt to reduce the risk of local recurrence in view of the possibility of microscopic seeding at surgery. There was also no role of adjuvant chemotherapy or radiotherapy in view of the known resistance of grade 1 chondrosarcoma.

Patient was discharged home on 7^th ^postoperative day. He has been followed at 3 monthly intervals with routine radiography including CT scans. He has made good recovery from the operation, is in NYHA class 1 and has shown no clinical or radiological signs of local recurrence, more than a year after his surgery.

## Discussion

Chondrosarcoma accounts for nearly 30% of all malignant bone neoplasms and is the second most common matrix producing malignant tumour of bone. It is more common in central sites such as pelvis, proximal long bones and bones of anterior chest wall which, in turn, makes radical surgery difficult. 75% of chest wall chondrosarcomas arise from costochondral arches or sternum. The tumor most commonly occurs in the 3^rd ^to 5^th ^decades of life and is more frequent in men. Most chondrosarcomas arise de novo, but causal relationship between benign cartilaginous tumors [1], trauma [2,3], fibrous dysplasia and Paget's disease [4] has been described. Most patients present with a painful, slowly enlarging mass which appears radiologically as a lobulated mass in the medulla of the bone, with poorly defined margins, cortical thickening or destruction depending on the grade of the tumour, mottled, flocculent calcified matrix, endosteal scalloping and, uncommonly, pathological fractures. Morphologically, chondrosarcomas are subclassified into conventional intramedullary and juxtacortical, clear cell, dedifferentiated [5] and mesenchymal variants. Conventional chondrosarcomas have lobules of hyaline and myxoid cartilage in the medullary cavity, growing through the cortex and forming a well defined circumcised mass. Grade 1 chondrosarcomas have only mild hypercellularity, infrequent mitotic activity and sparse binucleate cells. Grade 3 chondrosarcomas, on the other hand, demonstrate marked hypercellularity, frequent mitoses, extreme pleomorphism and bizarre tumour giant cells, seen more predictably with chondroblastic osteosarcoma. The clear cell chondrosarcomas have osteoclast type giant cells with clear cytoplasm with reactive bone formation mimicking osteosarcomas. Mesenchymal chondrosarcomas comprise islands of hyaline cartilage surrounded by sheets of small round cells making differentiation from Ewing's sarcoma or even hemangiopericytoma occasionally difficult. Mesenchymal variant as also the clear cell subtype are typically seen in younger patients. The dedifferentiated chondrosarcomas have a dedifferentiated component similar to a malignant fibrous histiocytoma or a fibrosarcoma in terms of its anaplastic content and aggressive behaviour [6].

Our patient was a 52 year old male, with no antecedent history of trauma, with no symptoms suggestive of the presence of chest wall chondrosarcoma, even when seen retrospectively. The mass was arising from the right costal margin and the adjacent ribs, but mostly growing into the soft tissues inwards towards the pleural cavity. The gross appearances of the mass including its cartilaginous texture, and its variable density with intervening solid and cystic areas, suggested either a teratoma or a chondrosarcoma. It was considered inappropriate to proceed with just the coronary artery surgery as initially planned in the presence of a tumour which could be malignant. A wide excision was therefore performed with in continuity excision of the chest wall muscle and periosteum but without resection of the ribs in view of the number of ribs involved. Frozen section suggested either a possibly malignant cartilaginous tumour or a teratoma. Since frozen section also suggested the possibility of teratoma, further resection of all the ribs, with whose periosteum the tumor was in contiguity, was considered excessive both in light of the number of ribs involved as well as the possibility of microscopic seeding having already taken place even if paraffin sections would later confirm a chondrosarcoma. The paraffin section, 12 days later, confirmed grade 1 chondrosarcoma with positive margins.

In view of the central location of chondrosarcomas, wide excision, including that of the bones and cartilages from which they arise, is not always possible. However, a chest wall chondrosarcoma is ideally treated by wide excision including that of the rib from which it arises. Excision of as many as 4 ribs has been reported [7] and a variety of materials have been used including double prolene mesh [8], tantalum mesh [9], bovine pericardium [10] and Marlex mesh with metal plates [7]. Chondrosarcomas are resistant to both chemotherapy and radiotherapy [11], although mesenchymal chondrosarcomas have been treated with wide excision and chemotherapy [12].

Coexistence of malignancy in patients undergoing surgery for coronary artery disease or valvular disease requires careful risk benefit analysis of various options available. Advanced, incurable malignancy associated with a limited prognosis may render surgical revascularisation inappropriate. A staged CABG procedure either before or after curative surgery for cancer is associated with advantages and disadvantages, determined in part by the organ involved by cancer and the urgency of the requirement for surgical revascularisation. In general, CABG before cancer surgery protects the patient from the risk of perioperative infarction during cancer surgery and confers a certain degree of haemodynamic stability during subsequent cancer surgery. It, however, delays the institution of potentially curative cancer surgery and subsequent chemotherapy or radiotherapy. It may also make cancer surgery technically more difficult if an intrathoracic cancer operation is needed. Simek et al, thus, reported carotid endarterectomy 2 months prior to aortic valve replacement and coronary artery bypass grafting followed 3 months later by successful meningioma excision in a 76 year old patient. [13]. Although concomitant surgery increases the duration of anaesthesia and surgery, and might result in higher bleeding complications owing to systemic heparinisation, it might quite often be the favoured option in view of the survival advantage of the earlier removal of a life threatening tumour.

Simultaneous planned excision of left atrial myxoma and CABG would seem obvious [[14] and [15]] but lung cancer has been frequently resected as a combined operation with CABG with no increase in either mortality or morbidity [16] including bilateral lung cancers though a median sternotomy [17]. Anatomic closeness of thymus lends it to concomitant excision along with coronary artery bypass grafting or other open heart procedures when affected by malignant disease. Ohshima et al reported combined CABG and excision of invasive thymoma [18], Kouzu et al reported CABG, aortic valve replacement and thymectomy for a mixed type thymoma [19] and Poullis et al described combined CABG and thymectomy for thymoma with pure red cell aplasia [20]. Successful off pump CABG has been described simultaneously with esophagectomy through left thoracoabdominal or transhiatal [21] approaches for oesophageal cancer, gastrectomy for gastric cancer [22] and Miles' operation for rectal cancer [23], with avoidance of heparinisation a distinct advantage in preventing bleeding complications. Both Litmathe et al [24] and Wolfhard et al [25] reported good results with simultaneous CABG and parathyroid resection for adenoma or simultaneous CABG and thyroidectomy whether for benign or malignant disease. Again, one stage surgical management of renal carcinoma complicated by inferior caval vein infiltration and coronary artery disease, with initial nephrectomy, excision of inferior vena caval and right atrial tumour under circulatory arrest followed by distal coronary anastomoses during rewarming have been successfully reported [26,27]. Presence of a concomitant phaeochromocytoma in a patient with coronary artery disease requires careful preoperative and intraoperative management of both pathologies in terms of blood pressure control, myocardial protection and haemostasis. To et al reported the first successful cases of combined coronary artery bypass grafting and laparoscopic adrenalectomy for phaeochromocytoma and excision of extra adrenal phaeochromocytoma [28]. Subsequent successes were described by Garg et al [29] and Balabaud-Pichon et al [30]. Chkuaseli et al described simultaneous successful coronary artery bypass grafting and panhysterectomy for endometrial carcinoma [31]. Similarly, successful concomitant coronary artery surgery has been described with curative excision of both male [32] and female [33] breast carcinoma. Chest wall tumours rarely coexist with coronary artery disease. Kostolny et al described extended resection of a chest wall desmoid tumour which had infiltrated inwards to involve a previous left internal mammary graft to left anterior descending coronary artery. The chest wall tumour along with LIMA was excised in continuity, a saphenous vein bypass graft fashioned off pump and chest reconstructed with polypropylene mesh and a latissimus dorsi musculocutaneous flap [34]. Rozhledy et al reported actinotherapy and hyperthermia followed by coronary artery bypass grafting, sternal resection and latissimus dorsi myocutaneous transposition in a patient with metastatic non differentiated carcinoma of sternum in whom the primary tumour was found neither preoperatively nor during a 36 month postoperative remission [35].

All the above, however, describe concomitant coronary artery surgery with excision of carcinomas affecting various organs of the body when the diagnosis of malignancy was made preoperatively. When malignancy is detected incidentally, for the first time, during a coronary artery bypass operation, or, indeed, any open heart operation, a quick, on the spot evaluation of the various management options and their risk benefit potential is required. Fortunately such a discovery is extremely rare during open heart surgery. Mirsadraee et al described the successful excision of a locally infiltrating asymptomatic incidentally discovered malignant thymoma along with coronary artery bypass grafting [36]. Abdullah et al described excision of a previously unsuspected thymic carcinoid during urgent CABG [37]. Guo et al cautioned that all enlarged internal thoracic lymph nodes during internal thoracic artery mobilisation should be sent for histopathology and reported three such incidental discoveries during LIMA mobilisation for CABG, two of the patients having previously undiagnosed lymphomas and the third a metastatic carcinoma of breast [38]. Walker et al suspected phaeochromocytoma in a patient undergoing coronary artery bypass grafting by extreme episodic spontaneous hypertensive episodes resulting in tearing of anastomoses, subsequently confirmed by laboratory findings and diagnostic imaging [39].

To conclude, chondrosarcoma of the chest wall may rarely coexist in a patient with coronary artery disease that requires surgical revascularisation. If the diagnosis of chondrosarcoma is made before CABG, the management, ideally, consists of excision of the tumour with a wide clearance including the ribs from which it arises, with reconstruction of chest wall with various synthetic meshes available. This could be done before, during or after coronary artery surgery depending on the urgency of revascularisation. If, however, the tumour is discovered incidentally during coronary surgery, the frozen section is not unequivocal and the tumour arises from a wide base necessitating an elaborate chest wall reconstruction, a wide resection, without excision of multiple ribs necessarily, and CABG, followed by careful surveillance for local recurrence is not inappropriate.

## Consent

Written informed consent was obtained from the patient for publication of this case report and accompanying images. A copy of the written consent is available for review by Editor-in-Chief of this journal.

## Competing interests

The authors declare that they have no competing interests.

## Authors' contributions

PK conceived, designed and drafted the manuscript and was the principal operating surgeon and consultant in charge of patient's care. DD was the anaesthetic consultant responsible for patient's anaesthetic and ICU care and made valuable suggestions. RR made the histopathological diagnosis and supplied the histopathology pictures. GS critically evaluated the manuscript and contributed intellectually.
